# Video-based formative and summative assessment of surgical tasks using deep learning

**DOI:** 10.1038/s41598-022-26367-9

**Published:** 2023-01-19

**Authors:** Erim Yanik, Uwe Kruger, Xavier Intes, Rahul Rahul, Suvranu De

**Affiliations:** 1 Department of Mechanical, Aerospace, and Nuclear Engineering, Center for Modeling, Simulation, and Imaging for Medicine (CeMSIM), Rensselaer Polytechnic Institute, Troy, 12180 USA; 2 Biomedical Engineering Department, Center for Modeling, Simulation, and Imaging for Medicine (CeMSIM), Rensselaer Polytechnic Institute, Troy, 12180 USA

**Keywords:** Biotechnology, Computer science, Scientific data, Statistics

## Abstract

To ensure satisfactory clinical outcomes, surgical skill assessment must be objective, time-efficient, and preferentially automated—none of which is currently achievable. Video-based assessment (VBA) is being deployed in intraoperative and simulation settings to evaluate technical skill execution. However, VBA is manual, time-intensive, and prone to subjective interpretation and poor inter-rater reliability. Herein, we propose a deep learning (DL) model that can automatically and objectively provide a high-stakes summative assessment of surgical skill execution based on video feeds and low-stakes formative assessment to guide surgical skill acquisition. Formative assessment is generated using heatmaps of visual features that correlate with surgical performance. Hence, the DL model paves the way for the quantitative and reproducible evaluation of surgical tasks from videos with the potential for broad dissemination in surgical training, certification, and credentialing.

## Introduction

The skill of the surgeon is the single most important determinant of the success of a surgical procedure^1^. Assessment of surgical skills may be formative or summative. Formative assessment is low-stakes. Experts typically provide it as guidance during surgery. On the other hand, summative assessment is employed in high-stakes certification or credentialing and is usually associated with a quantitative score computed by proctors. Though direct observation of surgeons in the operating room or on a simulator remains the current gold standard of surgical skill evaluation, video-based assessment (VBA) is receiving increasing attention^2–4^. The American Board of Surgery (ABS) is exploring VBA as a component of the Continuous Certification Program for general surgeons and related specialties^5^. However, as a post-hoc procedure, VBA is manual- and time-intensive, subjective, and prone to poor inter-rater reliability^2,3^. Moreover, VBA methodologies often entail editing the videos into snippets to reduce the workload^3^, promoting subjectivity due to the editor’s bias^2,3^. Further, numerous studies have reported inferior validity evidence and inflated score prediction via edited videos compared with complete videos^3^. Another limitation is that VBA is almost exclusively formative, i.e., low-stakes, and there is a notable gap in the literature regarding using VBA for summative, i.e., high-stakes, assessment^3^, such as Fundamentals of Laparoscopic Surgery (FLS). Hence, there is a need to develop an objective, efficient and automated approach for VBA.

Several deep learning (DL) models have been developed for automated and objective skill assessment^6^, most of which rely on obtaining sensor-based kinematics data from surgeons. This is time- and labor-intensive and may interfere with the surgical task. In contrast, videos are collected routinely as part of most surgical procedures^2^, making large-scale data collection feasible. Existing video-based DL models utilize editing to simplify the problem^7,8^. In addition, these models use label-preserving snippeting in which each snippet shares the label of the complete video. This is problematic as labels for the entire video may not apply to individual snippets. Finally, current DL models do not provide means to assess the salient features that characterize the performance. Explainable artificial intelligence (XAI) techniques^9,10^, such as class activation maps (CAMs)^11^, can address this^10,12^. Nevertheless, they have not been shown to provide formative evaluation reliably.

**Figure 1:**
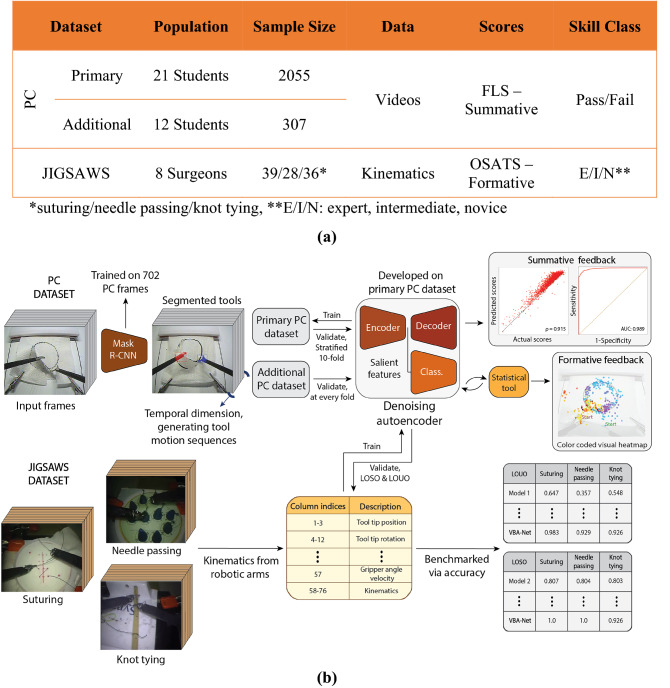
Overview of the study. **(a)** Subject demographics and descriptive data. **(b)** The pipeline of the VBA-Net. The model utilizes Mask R-CNN to generate tool motion sequences from video frames. Then denoising autoencoder (DAE) embeds the sequences for the classifier to predict summative and formative performance. The primary PC dataset is used to develop the model, i.e., tune its hyperparameters. The additional PC dataset, on the other hand, is used for validation. The JIGSAWS dataset is utilized to benchmark the model against the high-performing models in the literature.

To address these limitations, we propose a DL model, the Video-Based Assessment Network (VBA-Net), that can utilize complete surgical video sequences to provide summative surgical scores and generate formative feedback based on surgical performance. Figure 1 illustrates the overview of the study. Two datasets involving surgical pattern cutting (PC) were used to develop the VBA-Net (Fig. 1a). Further, to elucidate the generalizability of our model, we benchmarked it on the most commonly used public dataset, JIGSAWS^6,13^. Finally, we provided formative feedback via CAMs and presented a model-agnostic statistical tool to validate their saliency.

## Methods

### Dataset generation

There are two PC datasets in this study, namely primary and additional. PC is one of the five tasks of the FLS certification program, a prerequisite for board certification in general and ob/GYN surgery^14^. PC entails laparoscopic scissors to cut a circular pattern printed on a 10 cm × 10 cm gauze pad while applying traction with the Maryland Dissector (grasper). Both PC datasets were collected at the University at Buffalo, and all trials were executed in accordance with relevant guidelines and regulations approved by the Institutional Review Board (IRB) of the University at Buffalo and Rensselaer Polytechnic Institute. Further, subjects were informed of the experimental protocol and provided written informed consent for the study.

The primary PC dataset has 21 medical students (6 males and 15 females), ages between 21 to 30, with a mean age of 23.95, none of which has prior laparoscopy experience. In this dataset, the subjects executed the task for 12 days generating 2055 trials after the ones with negative scores were removed. On the first day, each subject executed the task once. Between days 2 and 12, subjects performed up to ten PC trials. Finally, on the last day, five repetitions were reported by each subject. The performance scores in FLS are high-stakes based on end-point metrics, e.g., time and precision error^15^. These scores categorize subjects into pass/fail classes (Table S1) based on a cut-off threshold^15^. Notably, this resulted in an imbalanced dataset where the pass/fail ratio is 8.9. The primary PC dataset was used to develop the model, i.e., select the hyperparameters.

On the other hand, the additional PC dataset has 12 subjects from an independent cohort, performing up to 26 times each in one day, generating 307 trials. We did not observe an imbalance in this dataset. This cohort was used to validate the model’s generalizability on unseen subjects. Notably, the videos were collected via the standard FLS box camera with 640 × 480 resolution at 30 FPS for both the PC datasets.

The JIGSAWS dataset^13^, on the other hand, contains sensor-based data collected via the da Vinci Surgical System (Intuitive Surgical, Inc) for tasks: suturing, needling passing, and knot tying^13^. The dataset has three surgical skill classes, viz., novice, intermediate, and expert, based on the hours spent in the operating room (OR). Moreover, modified Objective Structured Assessment of Technical Skills (OSATS) scores are available. OSATS is a formative assessment rubric^3^ computed based on low-stakes informative criteria^13,16^. In addition, global rating scales (GRS), a summation of individual items in the OSATS rubric, is available. This dataset was used to gauge the efficacy of the VBA-Net on different surgical tasks.

### Model development

#### Instance segmentation

Several studies have shown the effectiveness of video-based instrument tracking towards objective and automated assessment of skills^17–22^. Therefore, we used an instance segmentation network, Mask Region-based Convolutional Neural Network (Mask R-CNN) (For the architecture, see Fig. S3). Instance segmentation differs from object detection as the background is also a class in training and the model learns to segment the instance out of its background. This is beneficial when working on datasets with a constant camera view, e.g., the datasets used in this study, especially when background items such as clips resemble the surgical tools in use.

Mask R-CNN^23^ works by first extracting spatial features from the input frames using a CNN backbone, i.e., ResNet50. The spatial features are then processed in Region Proposal Network (RPN), generating regions of interest (RoI) for each instance. Here, RoI is assumed correct for detection confidences of 0.7 or higher. Next, RoIPool is applied to a third of the RoI to extract salient feature maps, and the RoIAlign algorithm is imposed to align the pre- and post-RPN features. Finally, the generated features are fed into the convolutional layers, outputting the class and the binary mask for each instance and the respective bounding box coordinates.

#### Generating tool motion sequences

Once trained, Mask R-CNN, ${f}_{m}\left(.\right)$, was used to generate bounding boxes for both the surgical tools at every frame in a given trial, i.e., ${{\varvec{K}}}_{i}=\left[{f}_{m}\left({x}_{i1}\right), \dots ,{f}_{m}\left({x}_{ij}\right),\dots {,f}_{m}\left({x}_{iT}\right)\right]\in {\mathbb{R}}^{TxD}$. Here, ${x}_{ij}$ is the j^th^ frame of the i^th^ trial in the dataset, and $T$ is the temporal length, i.e., number of frames. On the other hand, $D$ is the number of input features. $D$ is 4 in this study—Grasper and Scissor’s Cartesian coordinates. Finally, ${\varvec{K}}= \left[{K}_{1}, \dots ,{K}_{i},\dots ,{K}_{N}\right]\in {\mathbb{R}}^{Nx(TXD)}$ is the dataset of the tool motion sequences with N trials. Here, N is 2,055 and 307 for the primary and additional PC datasets. Notably for frames in which the model failed to detect the tools, i.e.,${f}_{m}\left({x}_{ij}\right)=\{\}$, the coordinates of the succeeding and preceding frames were averaged, as seen in Eq. (1).

1 $${f}_{m}\left({x}_{ij}\right)=\frac{({f}_{m}\left({x}_{ij-1})+ {f}_{m}\left({x}_{ij+1}\right))\right)}{2}$$

#### The denoising autoencoder

We extracted embedded features of the tool motion sequences via a denoising autoencoder (DAE) (For the architecture, see Fig. S4) with Gaussian noise (alpha = 0.001). The DAE is an unsupervised CNN-based autoencoder. Autoencoders have been used for surgical skills assessment in several studies^17,22,24,25^. DAE consists of an encoder to extract the important features (${{\varvec{K}}}_{{\varvec{e}}})$ from the noisy input and a decoder to reconstruct the input based on the features provided by the encoder. Here, ${{\varvec{K}}}_{{\varvec{e}}}= \sigma \left(\left[{K}_{1}, \dots ,{K}_{i},\dots ,{K}_{N}\right]\right)\in {\mathbb{R}}^{Nx(TX{D}_{e})}$. $\sigma$ is the output of the encoder and ${D}_{e}$ is the output number of features.

#### The residual classifier

Once the salient features, ${{\varvec{K}}}_{{\varvec{e}}}$, were extracted, we utilized a CNN-based classifier (Fig. S4), assessed summative skills, and provided formative feedback. We utilized an in-house attention-infused residual block to prevent the vanishing gradient problem^26^ for both the encoder and the classifier. Specifically, our residual block consisted of two identical convolutional layers and an identity layer. Moreover, two spatial and channel squeeze and channel excitation (scSE)^27^ attention layers were included for their ability to recalibrate the input feature maps by highlighting the most salient features in the residual block. The first scSE was placed between the initial and second convolutional layers. The second scSE was after the residual weights were added to the second convolutional layer. In addition, the convolutional layers within the residual block were dilated when training for classifier^28^.

When training the classifier, a Global Average Pooling (GAP)^29^ layer followed the residual block, aggregating the feature maps and feeding them to the fully connected layer while allowing training of the model with inputs of different sizes. Lastly, a fully connected layer consisting of one node and no activation when trained for regression and two nodes and Softmax activation for binary classification were added to output the FLS scores and skill classes, respectively.

### Model training

#### Mask R-CNN

We pre-trained Mask R-CNN on the COCO dataset^30^ and fine-tuned the classifier on frames from both PC datasets. Further, the output layer was configured to accommodate each class, i.e., scissors, grasper, and the background. 702 frames were randomly selected for training from all 2362 videos where both scissors and grasper are available. This is to optimize the coverage of conflicting scenarios during training. 562 (80%) frames are used to train and validate Mask R-CNN and 140 (20%) for testing. Among these 562 frames, 450 (80%) and 112 (20%) were used for training and validation, respectively. Further, all the frames were resized to 512 × 512 from 640 × 480. Finally, the VGG Image Annotator (VIA)^31^ was used to annotate scissors and grasper tooltips in each frame using polygon annotation, the standard input for Mask R-CNN^23^.

We trained only the heads of the (Mask R-CNN) for 40 epochs while keeping the remaining layers frozen. We augmented the frames by implementing Gaussian Blur (sigma = 0–5) and horizontal flipping 50% of the time per epoch.

#### The denoising autoencoder and the classifier

Before training the denoising autoencoder (DAE) and the classifier via the extracted motion sequences, ${\varvec{K}}$, each sequence was downsampled to 1 FPS to reduce training time^20^. Moreover, the sequences were normalized using min–max normalization. Lastly, the performance scores were pre-processed via z-normalization, and one hot encoding was used for the class labels. The same pre-processing pipeline as the PC datasets was used for the JIGSAWS dataset kinematics.

The batch size was one during training because each input has a different sequential length. The training was regulated using early-stopping based-on validation loss with the patience of 4 and 20 epochs for DAE and classifier training, respectively, for the PC datasets. These values were 40 and 200 for the JIGSAWS dataset^13^. Finally, we incorporated class weights into the training to account for imbalance. (For hyperparameter selection, see Supplementary Information / Hyperparameter selection).

Notably, when developing the VBA-Net on the PC datasets, we repeated the training for ten sessions, ensuring robust hyperparameter selection. The training was conducted on a workstation with AMD Ryzen 7 2700X and NVIDIA GeForce RTX 2070.

### Model cross-validation

#### Train/validation/test split

In this CV, the data is randomly divided into train, validation, and test folds. The training and validation folds are used to develop the model, i.e., the training split is used to compute the training loss, while the validation split is to compute the validation loss. Test fold is then used to test the trained model’s efficacy. This CV was used to train and validate the Mask R-CNN.

#### Stratified tenfold

The data is randomly divided into ten folds, with the class imbalance ratio preserved. Then one fold is used for testing, while the remaining nine train the model. This is repeated until every fold is used for testing. This CV scheme is useful in utilizing all the available data. In this study, we used this CV for training and validating the VBA-Net on the main and additional PC datasets.

To evaluate the benchmarked models, we employed the standard CV schemes for JIGSAWS, i.e., leave-one-supertrial-out (LOSO) and leave-one-user-out (LOUO).

#### Leave-one-supertrial-out (LOSO)

LOSO CV scheme is a specialized version of the k-fold CV used by the majority of the papers on the JIGSAWS dataset. In LOSO, the ith trial of each participant is used for testing, while the remaining trials are used for training the network. Thus, LOSO is advantageous in assessing the model’s performance on unseen data. However, it is specifically developed for the JIGSAWS dataset and has limited utility in the literature. Furthermore, LOSO is not informative for the cases where the model evaluates new surgeons. LOUO overcomes this limitation.

#### Leave-one-user-out (LOUO)

In LOUO, the trials of a single subject are removed from the training process and used to test the model. This is repeated for each subject. Therefore, the network is challenged to generalize to an unseen subject from a different cohort or distribution. Moreover, LOUO can be used with any dataset with more than one subject performing. The downside of LOUO is that it is blind to the model’s performance on unseen data of the same subject, a crucial element for training.

In stratified tenfold, LOSO, and LOUO CVs, the performance was computed based on the overall confusion matrix built by combining all the predictions from each fold.

### Model evaluation metrics

When generating tool motions, Mask R-CNN was evaluated via average precision with intersection over union (IoU) being 0.5 to consider the predicted bounding box true^23,30,32,33^. IoU is the overlap ratio between the ground truth and the predicted bounding boxes.

We employed the Spearman correlation coefficient *(ρ*) to evaluate the score prediction performance, whereas accuracy, sensitivity, specificity, and area under curve (AUC) of the Receiver Operating Characteristics (ROC) curve were used to assess the binary classification results. On the other hand, when benchmarking the VBA-Net on the multi-class JIGSAWS dataset, we employed accuracy to evaluate the classification results. In contrast, *ρ* was used for OSATS (*ρ*_*OSATS*_*)* and GRS (*ρ*_*GRS*_*)* score predictions where *ρ*_*OSATS*_ was the mean value of *ρ*s for every six OSATS subscores^9,13,34^.

#### Trustworthiness

Besides the commonly used metrics, we utilized the recently proposed trustworthiness metrics^35,36^, i.e., question–answer trust, trust density, conditional trust density, trust spectrum, and NetTrustScore (NTS), to assess the reliability of the VBA-Net on the classification results. In this concept, the Softmax probability is associated with confidence, C(y|x), and a model, *M*, is trustworthy when a true prediction is accompanied by stronger Softmax and vice versa. Equation (2) presents the question–answer trust.

2 $${Q}_{z}\left(x,y\right)=\left\{\begin{array}{c}{C(y|x)}^{\alpha }\\ 1-{C(y|x)}^{\beta }\end{array}\genfrac{}{}{0pt}{}{if x \epsilon { R}_{y=z}|M}{if x \epsilon { R}_{y\ne z}|M}\right.$$

In Eq. (2),$${R}_{y=z}$$ is the space of all the samples (x) for which the predicted ($$y)$$ and the actual ($$z)$$ classes match. On the other hand, $${R}_{y\ne z}$$ is the space where they do not. Next, $$\alpha$$ rewards confidence for true predictions, and $$\beta$$ penalizes over-confidence when the forecast is incorrect. In this study, both are set to 1. Finally, $${Q}_{z}\left(x,y\right)$$ denotes the question–answer trust for a given class (z).

Next, trust density is the probability density distribution of ${Q}_{z}\left(x,y\right)$ mapped via the non-parametric density estimation with a Gaussian kernel ^36^. Moreover, conditional trust density takes trust density one step further by calculating the distributions separately for when ${R}_{y=z}$ and ${R}_{y\ne z}$. It helps spot overconfidence and overcaution for a given class (z). As a remark, in binary classification, ${R}_{y=z}$ represents the True Positive (TP) or True Negative (TN) whereas ${R}_{y\ne z}$ represents the False Negative (FN) or False Positive (FP).

The trust spectrum, ${T}_{M}\left(z\right)$, represents the overall trust behavior based on every class and NTS (${T}_{M}$) is the overall trustworthiness score generated by integrating the trust spectrum, see Eq. (3).

3 $${T}_{M}\left(z\right)=\frac{1}{N}\int {Q}_{z}\left(x\right)dx$$

$${NTS (T}_{M})= \int P\left(z\right){T}_{M}(z)dz$$

Here, $N$ is the sample size for a given class.

### Class activation map (CAM)

CAM is a visualization tool highlighting the regions that contribute the most to the classification prediction. It is based on the Hadamard product of the pre-Softmax weights and the activations of the last convolution^11^. If ${f}_{k}(i)$ represents the activations at the convolutional layer preceding global average pooling (GAP) for the unit k and timestamp *i* and ${{w}_{k}}^{c}$ is the pre-softmax weights between the GAP layer and the fully-connected classifier for the same unit and class *c*; CAM is defined as follows:

4 $${M}_{c}\left(i\right)= \sum_{k}{{w}_{k}}^{c}{f}_{k}(i)$$

We utilized CAMs to provide formative feedback for each trial.

### Statistical analysis for formative feedback validation

First, we masked each input by element-wise multiplying them with their respective CAMs. Then we trained the VBA-Net again from scratch using the weighted inputs and evaluated it via the stratified tenfold CV. As a result, we ended with two distributions for the given metrics, e.g., accuracy: before-masking and after-masking, both of which have ten samples reflecting the selected CV scheme. Next, we employed a one-sided Wilcoxon sign test to check whether the mean of the distribution was significantly different for the after-masking scenario. Here, our null hypothesis, H_0_, presumed no significant difference, whereas the alternative hypothesis, H_1_, assumed that the mean of the distribution for the after-masking is significantly greater. The significance was 0.05 for this analysis.

## Results

### Performance of Mask R-CNN

Mask R-CNN successfully extracted bounding box centroids (X, Y) from the surrounding artifacts, e.g., mechanical clips, in challenging conditions such as overlapping tools and blurred frames (Fig. S1). It reported an average precision of 0.97 when the intersection over union (IoU) is 0.5. Notably, the false predictions, i.e., IoU < 0.5, were not due to the inaccurate positioning of the tools, which may negatively affect the tool sequence data. Instead, they were due to tools being partially out of the camera vision or occluded by the gauze.

### Performance on the primary PC dataset

**Figure 2:**
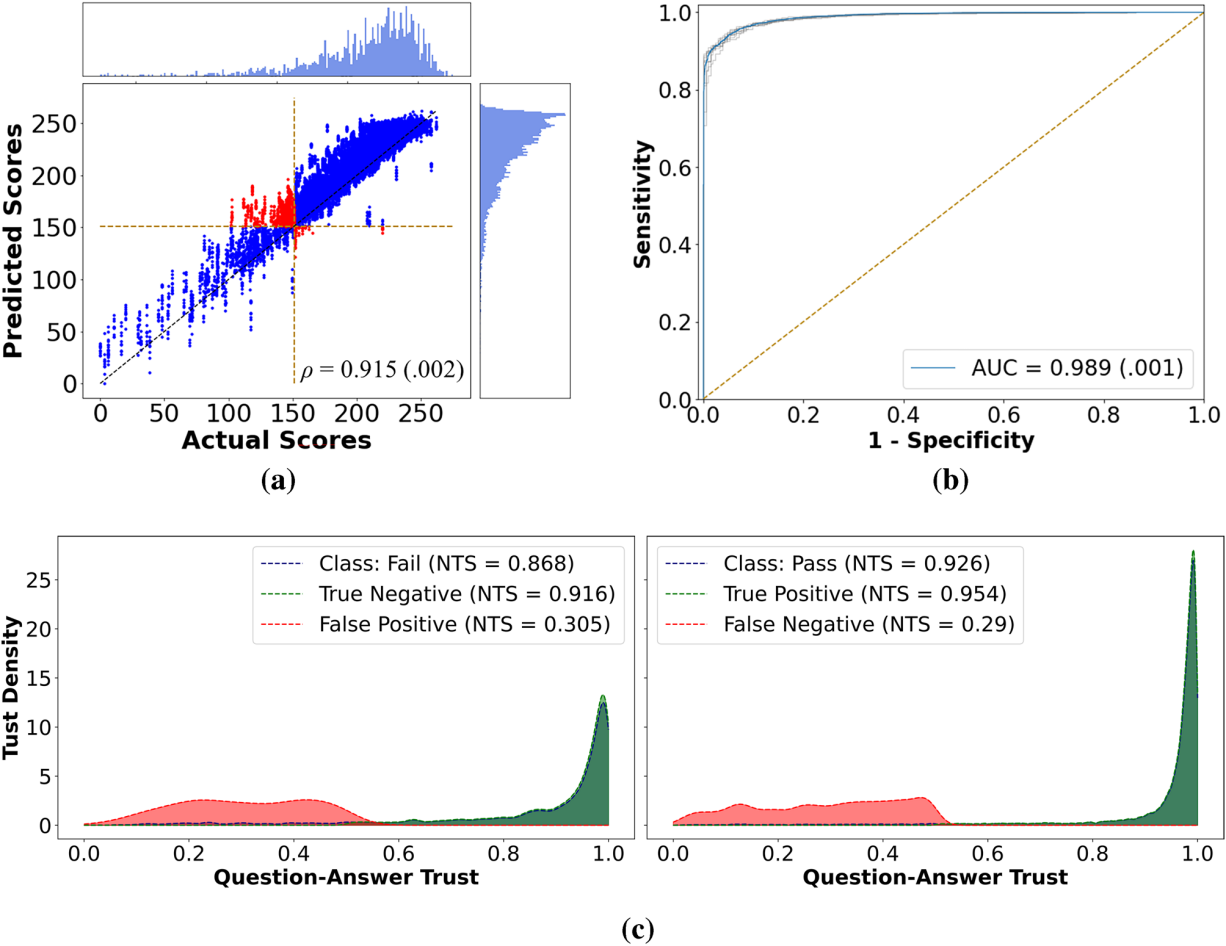
Results for the primary PC datasets. **(a)** Actual vs. predicted FLS scores for all ten training sessions combined. Here, the histograms show the frequency of samples for a given score. As seen, the network has a slightly inflated score prediction trend resulting in some trials close to the cut-off ratio to cross it—shown in red. Since classification analysis was conducted separately, this inflated prediction does not affect the pass/fail prediction accuracy. **(b)** The ROC curves. The blue line is the average of 10 running sessions, each shown in gray. The yellow line represents the random chances. **(c)** Question–answer trust plots for each class. The VBA-Net has high trustworthiness for true predictions. i.e., Softmax probabilities are close to 1.0 for the majority of the samples, as shown in green. On the other hand, the network is cautious about wrong predictions, i.e., the Softmax probabilities are close to the threshold of 0.5 and do not accumulate on the extreme end of 0.0—illustrated in red.

Using the salient features from the autoencoder, the classifier robustly predicted the FLS scores (Fig. 2a.) with an average *ρ* of 0.915 ± 0.002 after ten sessions with p < 0.05 for each. Moreover, VBA-Net achieves an accuracy of 0.955 ± 0.002 while reporting 0.958 ± 0.003 and 0.922 ± 0.010 for sensitivity and specificity, respectively. Further, the model has an area under the curve (AUC) of 0.989 ± 0.001 for the receiver operating characteristics (ROC) curve (Fig. 2b).

The model’s trustworthiness is analyzed in a single training session via trustworthiness metrics^35,37^. Figure 2c shows the trust spectrum accompanied by the NetTrustScore (NTS). The VBA-Net has robust trustworthiness with NTS values of 0.926 and 0.868 for the passing and failing classes. Moreover, for both the classes, the conditional NTS is above 0.9 when the prediction is true and around 0.3 when the prediction is false, implying that the VBA-Net has strong confidence in true predictions with low uncertainty while it can benefit from additional data for both classes^35^.

### Validation on the additional PC dataset

**Figure 3:**
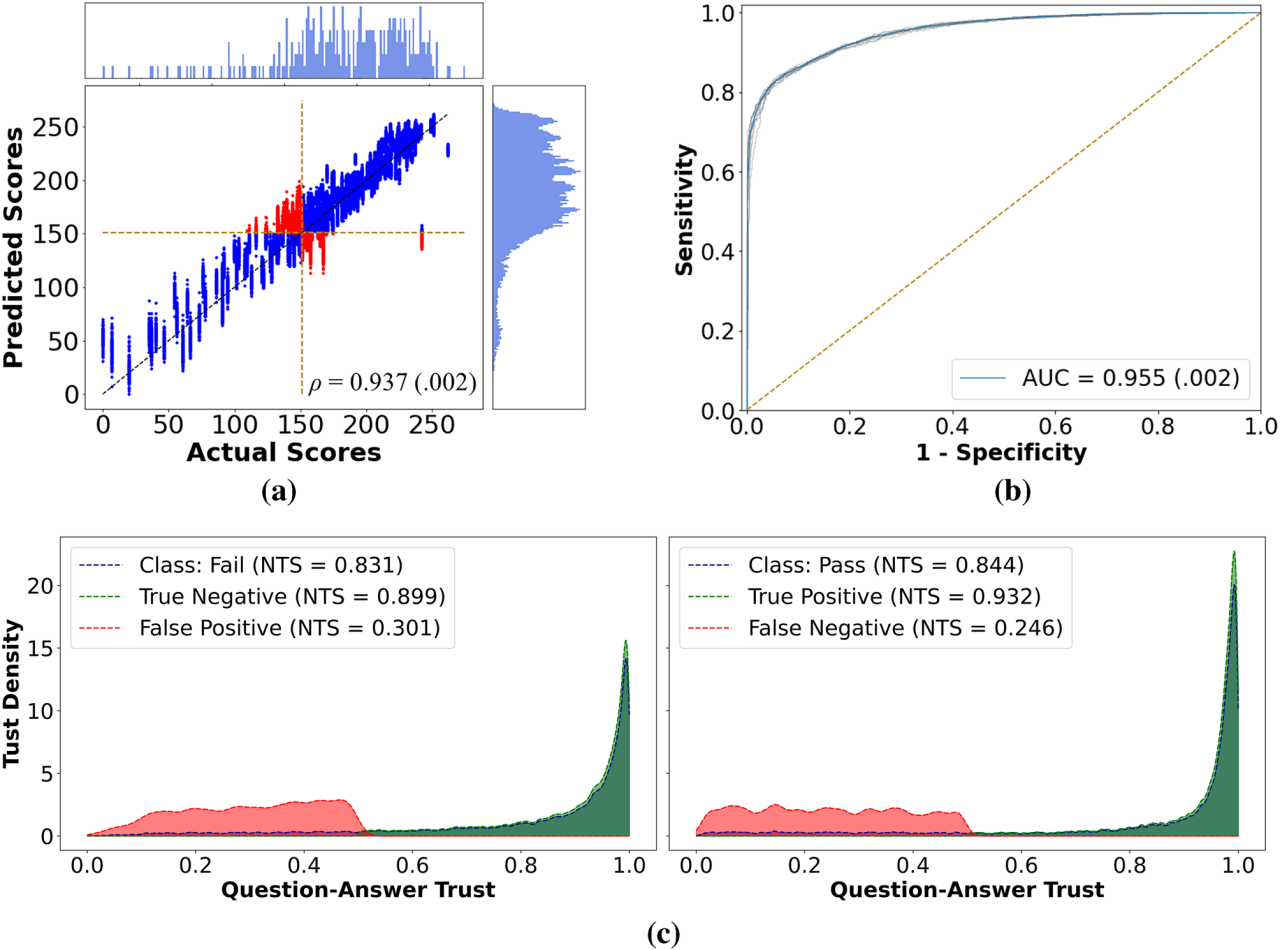
Results for the additional PC datasets. **(a)** Actual vs. predicted FLS scores for all ten runs. Here, we did not observe inflated score prediction, as shown in Fig. 2. This may be due to a more balanced representation of the samples. **(b)** The ROC curves. **(c)** Question–answer trust plots for each class. We observed the same confident true predictions and cautious wrong predictions trend in this plot compared to Fig. 2c.

For this analysis, we tested the VBA-Net, without retraining, on the additional PC dataset after every fold. This way, we could test the trained model’s performance on the unseen subjects, i.e., a different cohort. As a result, the VBA-Net surpassed its performance on the primary PC dataset it was trained on and successfully predicted the FLS scores (Fig. 3a) with *ρ* of 0.937 (with p < 0.05 for every fold). In addition, for classification analysis, VBA-Net reported an accuracy of 0.876 ± 0.002, with sensitivity and specificity of 0.871 ± 0.005 and 0.887 ± 0.11, respectively. Finally, the VBA-Net’s separability remained robust, with an AUC of 0.955 ± 0.002, as seen in Fig. 3b.

Figure 3c shows the trust spectrum accompanied by the NTS and the conditional NTS scores. VBA-Net manages low uncertainty and high trustworthiness reporting NTS values of 0.844 and 0.831 for the passing and failing classes. When Fig. 3c is compared with Fig. 2c, we see the VBA-Net retains its prediction confidence for true predictions, while for passing cases, it reports lower NTS for false predictions, indicating the need for additional data on passing cases for the additional PC dataset. This is expected as the additional PC dataset has 202 passing samples compared to 1842 for the primary PC dataset (Table S1).

### Validation on the JIGSAWS dataset

The generalizability of the VBA-Net on a separate task is demonstrated via leave-one-super-trial-out (LOSO) and leave-one-user-out (LOUO) CV schemes.

#### *Comparison *via* LOUO CV*

Based upon the LOUO CV scheme, the VBA-Net outperformed the current state-of-the-art results in all three surgical tasks, reaching the highest overall average classification performance (accuracy = 0.946; Table 1). Notably, VBA-Net realized perfect accuracy for experts for all the tasks in the JIGSAWS dataset while misclassifying only two novice trials: one for suturing and one for knot tying (Fig. S2a).

**Table 1:** Classification scores for LOUO CV.

Model	Method	Suturing	Needle passing	Knot tying	Mean
Zia and Essa^34^ [2018]	DCT	0.647	0.357	0.548	0.517
Zia and Essa^34^ [2018]	DFT	0.647	0.464	0.516	0.542
Fard et al*.*^40^ [2016]	M.F. + LR	0.705	–	–	–
Fard et al*.*^40^ [2016]	M.F. + SVM	0.721	–	–	–
Funke et al*.*^7^ [2019]	3DCNN	–	–	0.630	–
Zia and Essa^34^ [2018]	ApEn	0.882	0.857	0.774	0.838
Khalid et al*.*^25^ [2020]	Autoencoder	0.840	0.840	0.840	0.840
**VBA-Net**	**DAE + classifier**	**0.983**	**0.929**	**0.926**	**0.946**

*DCT* discrete cosine transform, *DFT* discrete Fourier transform, *LR* logistic regression, *SVM* support vector machine, *ApEn* approximate entropy, *M.F.* manual features.

Significant values are in bold.

In addition, VBA-Net reported the highest Spearman correlation coefficients for both OSATS and GRS prediction for all the tasks (Table 2), achieving a robust correlation for needle passing and knot tying while a moderate correlation for suturing. (For the breakdown of *ρ*, see Table S2).

**Table 2:** Regression scores for LOSO & LOUO CV.

Model	Suturing		Needle passing			Knot tying			Mean	
CV	LOSO	LOUO	LOSO		LOUO	LOSO		LOUO	LOSO	LOUO
Model 1	0.59|0.75	0.45|0.42	0.45|0.53		0.53|0.28	0.66|0.76		0.56|0.78	0.56|0.68	0.51|0.49
Model 2**	0.60|–	n/a	0.57|–		n/a	0.65|–		n/a	0.61|–	n/a
**VBA-Net**	**0.60|0.76**	**0.52|0.49**	**0.60|0.73**		**0.74|0.75**	**0.69|0.83**		**0.80|0.83**	**0.63|0.77**	**0.69|0.69**

At each cell, the format follows ρ_OSATS_|ρ_GRS._

Model 1: Zia and Essa^34^ [2018] and Model 2: Fawaz et al*.*^9^ [2019].

p > 0.05.

**Taken from respective authors’ papers but not included in the comparison.

Significant values are in bold.

#### Comparison via LOSO CV

Table 3 presents the LOSO CV results and corresponding benchmark models with at least 0.97 mean accuracy. (See Table S3 for results < 0.97). VBA-Net achieved perfect accuracy of 1.0 for suturing and needle passing tasks and provided an accuracy of 0.926 for the knot tying task, with a mean accuracy of 0.975, outperforming all the DL models (Fig. S2b). Here^9^, and ^10^ were not included in the analysis because, in their LOSO scheme, they further divided the training set into train and validation without providing the split ratio. This is different from the standard LOSO protocol^13^. Likewise^38^, was excluded as they utilized a fourfold (accuracy = 0.942) and tenfold CV (accuracy = 0.973), respectively. Notably, a machine learning (ML) model^34^ produces better mean accuracy than the VBA-Net. However, their approach is manually-intensive and not generalizable to other tasks.

**Table 3:** Classification scores (> 0.97) for LOSO CV and other CV schemes.

Model	Method	Suturing	Needle passing	Knot tying	Mean
Khalid et al*.*^25^ [2020]	Autoencoder	0.970	0.970	0.970	0.970
Nguyen et al*.*^41^ [2019]	CNN-LSTM	0.984	0.984	0.948	0.972
Fawaz et al*.*^9,12^* [2019]	CNN (adjusted LOSO)	1.0	1.0	0.921	0.974
Funke et al*.*^7^ [2019]	3DCNN + TSN	1.0	0.964	0.958	0.974
Soleymani et al.^38^* [2021]	CNN + FFT (tenfold)	N/A	N/A	N/A	0.973
**VBA-Net**	**DAE + classifier**	**1.0**	**1.0**	**0.926**	**0.975**
Castro et al*.*^10^* [2019]	CNN (adjusted LOSO)	0.984	0.989	0.989	0.987
Zia and Essa^34^ [2018]	ApEn**	1.0	1.0	0.999	0.999

*TSN* temporal segment networks, *FFT* fast Fourier transform.

*Taken from respective authors papers but were not included in the comparison.

**Approximate Entropy.

Significant values are in bold.

Moreover, the mean *ρ*_*OSATS*_ and *ρ*_*GRS*_ were 0.63 and 0.77 for the LOSO CV, exceeding the state-of-the-art performance (Table 2). The VBA-Net outperformed the existing models in OSATS score prediction on all three tasks. For GRS prediction, on the other hand, the VBA-Net achieved the highest performance for each task. (For the breakdown of *ρ*, see Table S4).

### Formative feedback

#### Heatmaps

**Figure 4:**
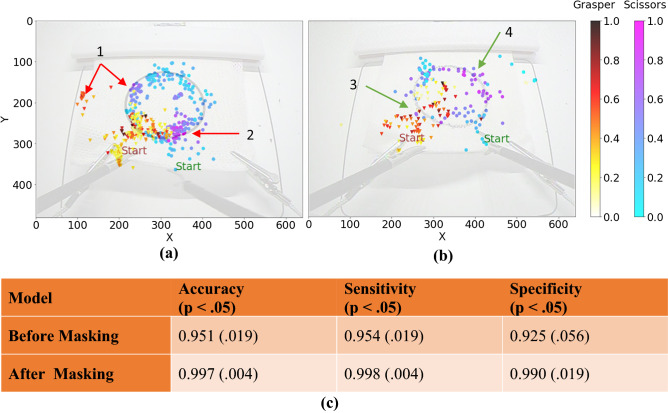
CAM results. CAM plots for **(a)** a TN (FLS score: 16.8) and **(b)** a TP (FLS score: 170.7) sample. The plots are presented in the original frame size of 640 × 480. Each dot represents the tool location for a timestamp generated at 1 FPS. This resulted in 256 dots for the TN case as the procedure took 256 s and 105 for TP. The red arrows indicate tool motions that may lead to poor performance, while the green arrows indicate smooth behavior. The color-coded heatmaps illustrate the intensities of the same CAM generated for the given samples. However, different color maps are used for scissors and grasper locations. **(c)** Overall VBA-Net performance comparison before and after masking. Here, p is the p-value of the statistical analysis, and the numbers within the parentheses in the second and third rows represent standard deviation based on tenfolds of training.

This section analyses how VBA-Net provides formative feedback via a *post-hoc* explainability tool, i.e., CAM. Figure 4 shows the 2D CAMs projected onto the tool trajectory using a 1D color-coded contour for a TP (pass) (Fig. 4a) and a TN (fail) (Fig. 4b) case.

In Fig. 4a, we provide an example of a TN case (fail) and annotate (red) the locations corresponding to poor performance based on the surgical videos. The subject started smoothly, successfully reaching the circular pattern from the corner of the gauze without unnecessary movement. However, they failed to cut the first half of the circle after multiple attempts and eventually moved to the second half without completing the first half. The high activation pointed out by ‘arrow 1’ captures this behavior. Simultaneously, the grasper was repositioned from the lower-left corner of the gauze to the middle left, a move that was not observed in the passing cases. The subject struggled through the second half, failing to cut the circle while holding the gauze with the grasper. The high activation at ‘arrow 2’ captures this.

We also analyzed a TP case (pass) in Fig. 4b. Here, we annotated the desired performance (green) based on the corresponding video. As a result, we observed that the subject uses the grasper effectively and avoids unnecessary grip attempts (arrow 3). Moreover, the subject uses smooth motion and cuts the gauze fast when cutting the second half of the circular pattern. The network captures this desired behavior, as pointed out by ‘arrow 4’. Overall, we observed that the activations are independent of the duration and are specific to each trial.

#### Statistical analysis results

To establish the effectiveness of such formative assessment without expert guidance, we analyzed CAMs via a model-agnostic statistical tool. We hypothesized that if CAMs highlight the salient parts, the model should distinguish better between skill classes when the input sequences are masked with CAM. Consequently, the results should improve. We implemented our approach on the primary PC dataset in a single training session with a stratified tenfold CV. Resulting training, we obtained a distribution of metrics for each fold before and after masking. When comparing the distribution’s mean for each metric (Fig. 4c), the after-masking case achieved significantly greater performance than the before-masking case.

## Discussion

VBA has garnered significant attention for surgical skill assessment following the shift to competency-based medical education and patient safety. It promises to enhance the formative assessment of the learning process by offering trainees timely feedback while also allowing experienced surgeons to reflect on their surgical techniques. However, VBA methods need to be scalable, generalizable, and demonstrate a high level of correlation with current summative methods employed in the field. Herein, we demonstrated that VBA-Net offers excellent and trustworthy performances in various surgical procedures. The performance metrics presented in the previous section underscore the effectiveness of the VBA-Net in objective and automated summative score prediction.

VBA-Net can generalize well to unseen data. Thus, it can help individual trainees prepare for high-stakes certification exams such as FLS by providing reproducible scores in real time. Moreover, VBA-Net generalizes well to unseen subjects. Hence, it can assist proctors with the certification process as each subject performs one time and receives an end-point result. Besides, the model yields solid binary classification performance, particularly for specificity, i.e., the model was robust in detecting false certification for both unseen data and subjects. This finding is important as human error is one of the leading causes of death in the OR; hence poor clinical outcomes^39^ and preventing false certification can significantly reduce that. In summary, these attributes can significantly contribute to more robust validity evidence, i.e., improving patient outcomes.

We validated the generalizability of the VBA-Net by benchmarking it against the state-of-the-art models on the JIGSAWS dataset. Based on the LOUO CV, the VBA-Net improved the average OSATS and GRS score predictions by 35.3% and 40.8%. Further, VBA-Net outperformed the closest ML model^34^ with a 12.9% margin and the closest DL model^7^ in knot tying with a 47% margin in classifying the surgeons. This shows that the VBA-Net can generalize to tasks other than PC and can predict OSATS scores of new subjects.

In addition, for unseen trials measured via LOSO, VBA-Net achieved the highest Spearman correlation coefficient in predicting both the OSATS and GRS scores, indicating that the VBA-net can predict the performance on the unseen trials better, supporting proctoring of the trainees. Here, the model reported comparatively lower accuracy in knot tying for classification analysis. We can attribute this to the complexity of the knot tying task, as stated in the literature^7,12^. Besides, when comparing LOUO with LOSO, we observed a decrease in classification and regression performances, signifying that the subjects demonstrated class-specific bimanual motor behavior. Finally, we noticed that several studies^7,9,10,12,34^, including ours, reported perfect accuracy on Suturing and Needle Passing tasks via LOSO. Therefore, we believe the field can benefit from new publicly available surgical datasets.

Now we discuss the formative feedback. VBA-Net successfully highlighted the parts of the procedures that separated the performance in the Pass and Fail classes, as seen in Fig. 4a,b. Moreover, the statistical analysis showed a significant improvement in the VBA-Net’s performance when CAM plots were used to mask the input sequences (Fig. 4c). These results signify that the CAMs are valid and highlight the essential parts of the sequence toward the skill class. Hence they can be used for low-stakes informative feedback. Moreover, such visual maps can draw the proctor’s attention to the distinct parts of the videos, thus improving the time-effectiveness of the assessment, i.e., it can reduce the workload and burnout, an important restriction to VBA. Moreover, these validated maps can lead to objective and automated editing to establish time-efficient and generalizable low-stakes rubrics for surgical education.

Still, our study has several limitations. First, tool trajectories are the only extracted features from the videos; hence, while the literature is well-established on tool tracking, whether it is the optimal feature set remains unclear. Second, our model is not end-to-end. This has its strength in using either videos or kinematics as inputs, but it increases the framework’s complexity which could be reduced using an end-to-end model. Finally, we aim to overcome these limitations by developing end-to-end video-based DL pipelines for surgical skill assessment.

## Conclusion

We have developed a state-of-the-art DL pipeline—VBA-Net—that is trustworthy and can predict summative FLS scores and skill classes using entire surgical videos while providing statistically-verified formative visual feedback. We believe the VBA-Net has the potential for objective and real-time VBA of surgical skills in surgical training, certification, and credentialing.

## Supplementary Information

Supplementary Information.

## Data Availability

The PC dataset utilized in this study is not publicly available. The FLS scoring used on this dataset is disclosed to the *Center for Modeling, Simulation, & Imaging in Medicine (CeMSIM)* only under a Nondisclosure Agreement with the FLS committee. The benchmark dataset, JIGSAWS, is publicly available at: https://cirl.lcsr.jhu.edu/research/hmm/datasets/jigsaws_release/.
