# Supplementary material for: Video-based formative and summative assessment of surgical tasks using deep learning
Source: Sci Rep. 2023 Jan 19;13:1038. doi: 10.1038/s41598-022-26367-9 (PMC9852463; doi:10.1038/s41598-022-26367-9)
Supplement: Supplementary file 1 — Supplementary Information. [file 41598_2022_26367_MOESM1_ESM.pdf]

Supplementary Materials for

Video-based Formative and Summative Assessment of Surgical Tasks using Deep Learning

Erim Yanik[1], Uwe Kruger[1], Xavier Intes[1], Rahul Rahul[1], and Suvranu De[1*]

[1] Department of Mechanical, Aerospace, and Nuclear Engineering/Center for Modeling, Simulation, and Imaging for Medicine (CeMSIM)/Rensselaer Polytechnic Institute/Troy/12180.

[2] Biomedical Engineering Department/Center for Modeling, Simulation, and Imaging for Medicine (CeMSIM)/Rensselaer Polytechnic Institute/Troy/12180.

Supplementary Information

Hyperparameter Selection. During training the Mask R-CNN, the learning rate is initiated as 0.001, and the weight decay and momentum are 0.0003 and 0.9, respectively. The learning rate decreased by a factor of ten after the 20th epoch.

To extract salient features from motion sequences via the DAE, binary cross-entropy is minimized with a learning rate of 0.001. On the other hand, the classifier is trained with a mean squared error loss function when predicting the FLS scores and cosine similarity when performing classification, as it is shown to provide superior results on datasets with a limited sample size when trained from scratch[1]. The learning rate is 0.0002. Further, an Adam optimizer is used when training the model. Finally, the Scaled Exponential Linear Unit (SELU)[2] activation function is used for all the convolutional layers unless stated otherwise in Fig. S4. Finally, L2 regularizing (0.00001) is applied as both the kernel and activity regularizer[2].

Supplementary Figures

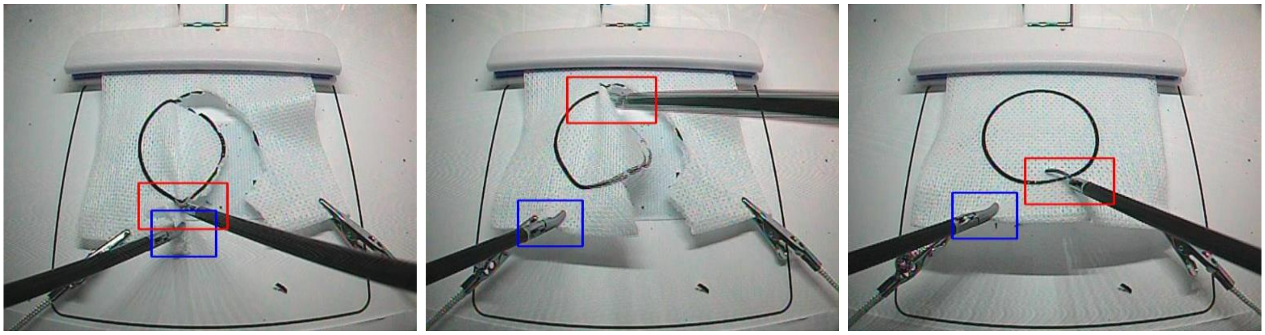

Fig. S1. Mask R-CNN output. Sample regressed bounding boxes of the tools. It is observed that the Mask R-CNN can successfully locate the tools when there is an intersection (left), blurry image (middle), and different tool angles (right).

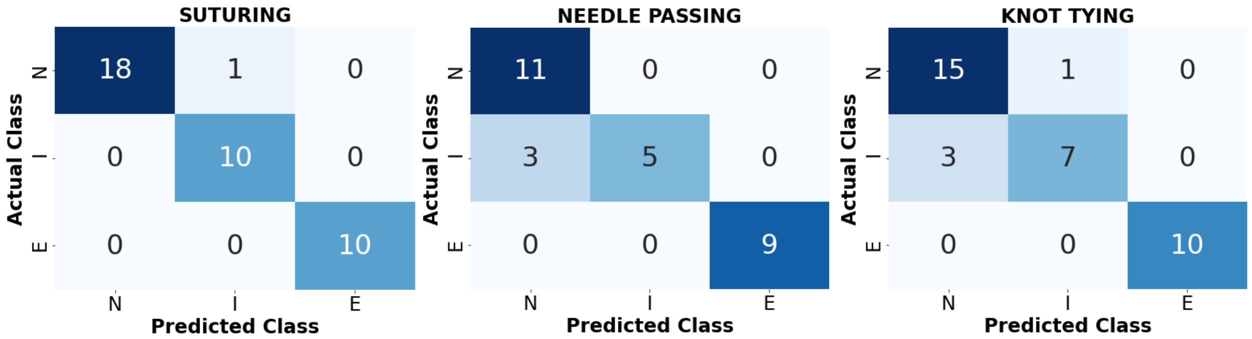

(a)

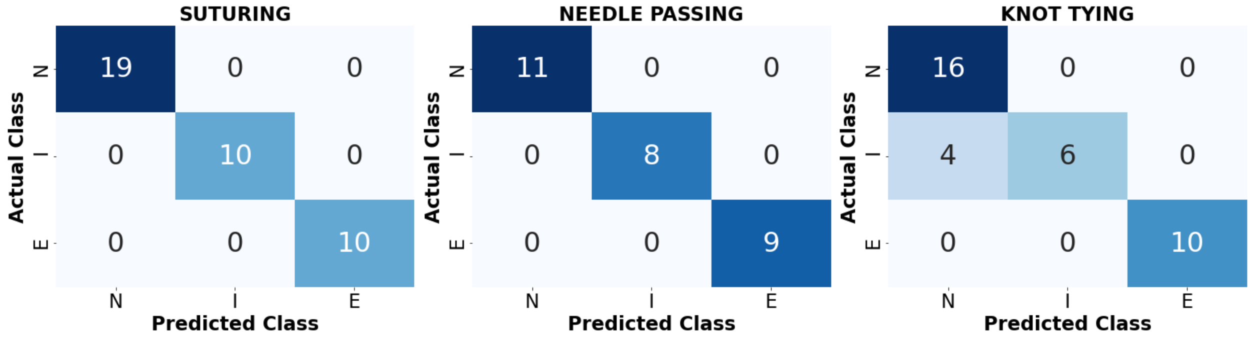

(b)

Fig. S2. Confusion matrices for surgical datasets. (a) For the JIGSAWS dataset via the LOUO CV scheme. Here, N, I, and E stand for Novice, Intermediate, and Expert, respectively. **(b)** For the JIGSAWS dataset via the LOSO CV scheme.

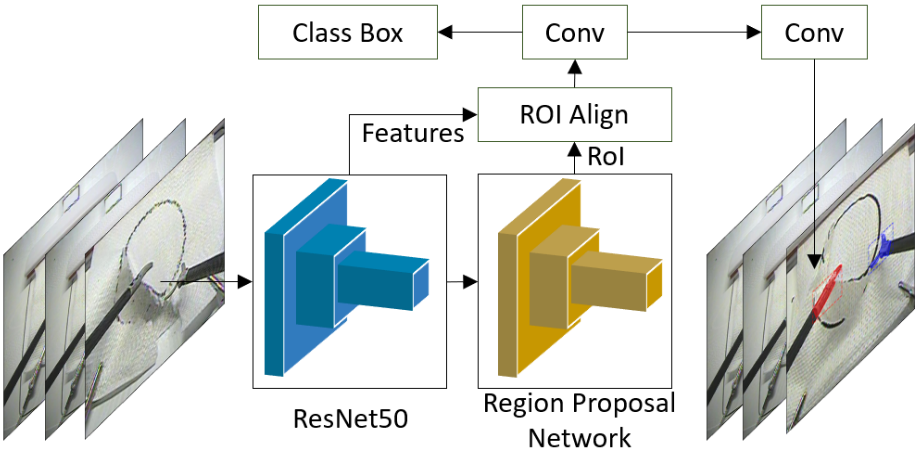

Fig. S3. Mask R-CNN architecture. It consists of a CNN-backbone, ResNet50, and Region Proposal Network (RPN) to output object properties.

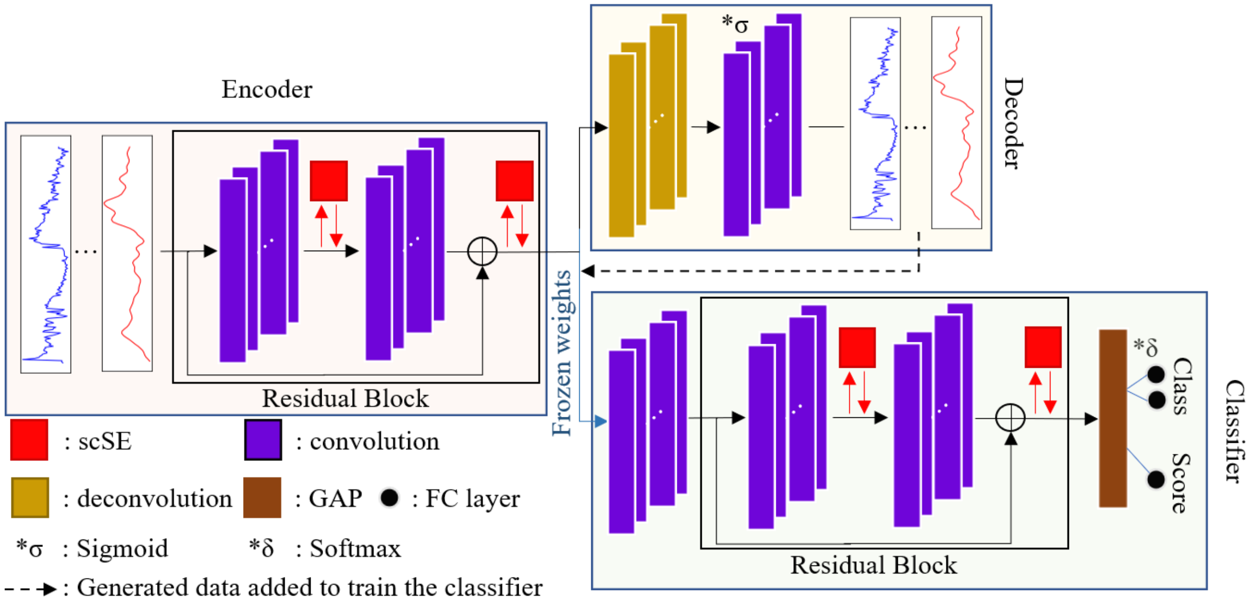

Fig. S4. The DAE and classifier structure. The figure illustrates the encoder, decoder, and classifier in their utilized order.

Supplementary Tables

Table S1. FLS score statistics based on binary classes for the PC datasets.

Dataset		No. of samples	Mean Duration (sec)	STD Duration (sec)	Mean FLS score	STD FLS score
**Main**	**Pass**	1842	73.5	25.0	208.3	24.3
	**Fail**	213	161.9	36.9	115.3	33.7
	**Overall**	2,055	82.7	37.8	198.7	38.1
**Additional**	**Pass**	202	86.0	23.4	185.2	19.3
	**Fail**	105	148.5	33.0	121.8	31.0
	**Overall**	307	107.4	40.2	163.5	38.5

Table S2. Breakdown of Spearman correlation coefficients, ρ_S, for OSATS scores prediction via the LOUO CV.

Task	Respect for tissue	Suture/needle handling	Time and motion	Flow of operation	Overall performance	Quality of final product
**ST**	$0.25^{\wr}$	0.67	0.66	0.59	0.53	0.43
**NP**	0.69	0.70	0.89	0.76	0.72	0.68
**KT**	0.75	0.78	0.86	0.78	0.76	0.87

$^{\wr}p > 0.05$. ST: suturing, NP: needle passing, KT: knot tying.

Table S3. Classification scores (<0.97) for LOSO CV and other CV schemes.

Author	Method	Suturing	Needle Passing	Knot Tying	Mean
Lajko *et al.*[3] [2021]	CNN	0.807	0.797	0.804	0.803
Anh *et al.*[4] [2020]	Autoencoder	0.835	0.823	0.806	0.821
Lajko *et al.*[3] [2021]	CNN + LSTM	0.816	0.832	0.828	0.825
Lajko *et al.*[3] [2021]	ResNet	0.819	0.842	0.835	0.832
Anh *et al.*[4] [2020]	LSTM	0.951	0.915	0.896	0.921
Wang and Fey[5] [2018]	CNN	0.925	0.954	0.913	0.931
Anh *et al.*[4] [2020]	CNN-LSTM	0.964	0.934	0.910	0.936
Anh *et al.*[4] [2020]	LSTM	0.965	0.941	0.912	0.940
Soleymani *et al.*[6] [2021]	CNN + FFT (4-fold)	N/A	N/A	N/A	0.942
Anh *et al.*[4] [2020]	CNN	0.968	0.954	0.927	0.950
Wang and Fey[7] [2018]	CNN-GRU	N/A	N/A	N/A	0.960
VBA-Net	***DAE + Classifier***	***1.0***	***1.0***	***0.926***	***0.975***

DAE: Denoising autoencoder, FFT: Fast Fourier Transform, HMM: Hidden Markov Model

Table S4. Breakdown of Spearman correlation coefficients, ρs, for OSATS score prediction via the LOSO CV.

Task	Respect for tissue	Suture/needle handling	Time and motion	Flow of operation	Overall performance	Quality of final product
**ST**	0.51	0.63	0.64	0.57	0.58	0.68
**NP**	0.52	0.77	0.65	0.69	0.55	0.43
**KT**	0.68	0.65	0.76	0.59	0.80	0.64

ST: suturing, NP: needle passing, KT: knot tying.

References

1. Barz, B. & Denzler, J. Deep learning on small datasets without pre-training using cosine loss. *Proc. - 2020 IEEE Winter Conf. Appl. Comput. Vision, WACV 2020* 1360–1369 (2020). doi:10.1109/WACV45572.2020.9093286

2. Castro, D., Pereira, D., Zanchettin, C., MacEdo, D. & Bezerra, B. L. D. Towards Optimizing Convolutional Neural Networks for Robotic Surgery Skill Evaluation. *Proc. Int. Jt. Conf. Neural Networks* **2019-July**, 1–8 (2019).

3. Lajko, G., Elek, R. N. & Haidegger, T. Endoscopic Image-Based Skill Assessment in Robot-Assisted Minimally Invasive Surgery. *Foot Ankle Spec.* **14**, 153–157 (2021).

4. Anh, N. X., Nataraja, R. M. & Chauhan, S. Towards near real-time assessment of surgical skills: A comparison of feature extraction techniques. *Comput. Methods Programs Biomed.* **187**, 105234 (2020).

5. Wang, Z. & Majewicz Fey, A. Deep learning with convolutional neural network for objective skill evaluation in robot-assisted surgery. *Int. J. Comput. Assist. Radiol. Surg.* **13**, 1959–1970 (2018).

6. Soleymani, A. *et al.* Surgical Skill Evaluation from Robot-Assisted Surgery Recordings. *2021 Int. Symp. Med. Robot. ISMR 2021* 1–6 (2021). doi:10.1109/ISMR48346.2021.9661527

7. Wang, Z. & Fey, A. M. SATR-DL: Improving Surgical Skill Assessment and Task Recognition in Robot-Assisted Surgery with Deep Neural Networks. *Proc. Annu. Int. Conf. IEEE Eng. Med. Biol. Soc. EMBS* **2018-July**, 1793–1796 (2018).
